# Supplementary material for: The RgaS-RgaR two-component system promotes Clostridioides difficile sporulation through a small RNA and the Agr1 system
Source: bioRxiv. 2023 Jun 27:2023.06.26.546640. Preprint. [Version 1] doi: 10.1101/2023.06.26.546640 (PMC10327067; doi:10.1101/2023.06.26.546640)
Supplement: Supplement 1 [file NIHPP2023.06.26.546640v1-supplement-1.pdf]

bioRxiv preprint doi: https://doi.org/10.1101/2023.06.26.546640; this version posted June 27, 2023. The copyright holder for this preprint (which was not certified by peer review) is the author/funder, who has granted bioRxiv a license to display the preprint in perpetuity. It is made available under aCC-BY 4.0 International license.

## SUPPORTING INFORMATION

**Figure S1. CRISPRi knockdown of *rgaS* and *rgaR* decreases *rgaS* and *rgaR* transcript levels.** Transcript levels of *rgaS* (**A**) and *rgaR* (**B**) at $H_{12}$ in 630Δ*erm* psgRNA-neg (MC2065), 630Δ*erm* psgRNA-*rgaS* (MC2066), and 630Δ*erm* psgRNA-*rgaR* (MC2227) grown on 70:30 agar supplemented with 2 μg/ml thiamphenicol and 1 μg/m nisin, as indicated. The means and standard error of the means of at least three independent biological replicates are shown. *, P < 0.01 by a Student's *t*-test.

**Figure S2. PCR confirmation of *rgaS* and *rgaR* mutants.** PCR verification of allelic replacement of *rgaS* (*CD0576*) and *rgaR* (*CD3255*) with the spectinomycin (*aad9*) or erythromycin (*ermB*) cassette, respectively in 630Δ*erm*, 630Δ*erm rgaS* (MC2228), and 620Δ*erm rgaR* (MC2229). Expected PCR products' sizes are: 3319 bp for the wildtype *rgaS* allele and 3078 bp for the Δ*rgaS*::*aad9* allele (primers oMC3123/3124); 2668 bp for the wildtype *rgaR* allele and 3436 bp for the Δ*rgaR*::*ermB* allele (primers oMC3261/3262).

**Figure S3. Toxin production is unaffected in *C. difficile rgaS* and *rgaR* mutants.** ELISA of TcdA and TcdB present in the supernatant of 630Δ*erm*, 630Δ*erm* Δ*rgaS* (MC2228), and 630Δ*erm* Δ*rgaR* (MC2229) grown in TY medium, pH 7.4, at $H_{24}$. The means and standard error of the means of four independent biological replicates are shown. The statistical analysis used for these data sets was a one-way ANOVA followed by Dunnett's multiple comparisons test.

**Figure S4. CRISPRi knockdown of RgaR-dependent genes specifically and significantly decreases their transcript levels.** Transcript levels of *CD0587*, *CD2098*,

bioRxiv preprint doi: https://doi.org/10.1101/2023.06.26.546640; this version posted June 27, 2023. The copyright holder for this preprint (which was not certified by peer review) is the author/funder, who has granted bioRxiv a license to display the preprint in perpetuity. It is made available under aCC-BY 4.0 International license.

*CD15111*, and *n00620* at $H_{12}$ in 630Δ*erm* expressing psgRNA-*neg* (MC2065) or CRISPRi targets for *CD0587* (MC2269), *CD2098* (MC2270), *CD15111* (MC2271), or *srsR* (MC2272) grown on 70:30 agar supplemented with 2 µg/ml thiamphenicol and 1 µg/m nisin. The means and standard deviations of three independent biological replicates are shown. *, P < 0.0001 by a Student's *t*-test.

**Figure S5. PCR confirmation of *srsR-CD16671* and *CD16671* mutants and *srsR* transcript length.** (**A, B**) PCR verification of allelic replacement of the *srsR-CD16671* locus with the spectinomycin (*aad9*) cassette in 630Δ*erm,* 630Δ*erm* Δ*srsR-CD16671* (MC2351; **A**) and 630Δ*erm* Δ*CD16671* (MC2363; **B**). Expected PCR products' sizes are: 2165 bp for the wildtype *srsR-CD16671* allele, 2745 bp for the Δ*srsR-CD16671*::*aad9* allele, and 3033 bp for the Δ*CD16671*::*aad9* allele (primers oMC3457/3458). (**C**) Primers spanning the regions within the *srsR-CD16671* locus depicted in (**D**), were used to amplify cDNA and RNA (minus reverse transcriptase negative control; -RT) from 630Δ*erm* grown on 70:30 agar at $H_{12}$ and 630Δ*erm* genomic DNA (gDNA).

**Figure S6. PCR confirmation of 630Δ*erm* *agrB1D1* mutants and R20291 *CDR20291_0503* (*rgaS*) and *CDR20291_3113* mutants (*rgaR*).** (**A**) PCR verification of allelic replacement of the *agrB1D1* locus with the spectinomycin cassette (*aad9*) in 630Δ*erm* and 630Δ*erm* Δ*agrB1D1* Expected PCR sizes are 2585 bp for the wild-type allele and 2930 bp for the *agrB1D1*::*aad9* allele (primers oMC3360/3361). (**B**) PCR verification of allelic replacement of *rgaS* and *rgaR* with the spectinomycin (*aad9*) cassette in R20291, R20291 Δ*rgaS* (MC2378), and R20291 Δ*rgaR* (MC2379). Expected PCR products' sizes are: 3215 bp for the wildtype *rgaS* allele and 2974 bp for the

bioRxiv preprint doi: https://doi.org/10.1101/2023.06.26.546640; this version posted June 27, 2023. The copyright holder for this preprint (which was not certified by peer review) is the author/funder, who has granted bioRxiv a license to display the preprint in perpetuity. It is made available under aCC-BY 4.0 International license.

Δ*rgaS*::*aad9* allele (primers oMC3124/3554); 2667 bp for the wildtype *rgaR* allele and 3056 bp for the Δ*rgaR*::*aad9* allele (primers oMC3261/3262).

**Figure S7. DNA cloning and vector details.**

**Table S1. RNA-seq of *rgaR* mutant compared to 630Δ*erm*.**

**Table S2. Bacterial strains and plasmids.**

**Table S3. Oligonucleotides.**
